# Reconstruction of the full-length transcriptome of cigar tobacco without a reference genome and characterization of anion channel/transporter transcripts

**DOI:** 10.1186/s12870-021-03091-6

**Published:** 2021-06-29

**Authors:** Hui Zhang, Jingjing Jin, Guoyun Xu, Zefeng Li, Niu Zhai, Qingxia Zheng, Hongkun Lv, Pingping Liu, Lifeng Jin, Qiansi Chen, Peijian Cao, Huina Zhou

**Affiliations:** 1 China Tobacco Gene Research Center, Zhengzhou Tobacco Research Institute of CNTC, 450000 Zhengzhou, China; 2 Haikou Cigar Research Institute of China National Tobacco Corporation, Hainan Province 570000 Haikou, China

**Keywords:** Cigar tobacco, Full-length transcriptome, RNA-Seq, Anion channels/transporters

## Abstract

**Background:**

Cigar wrapper leaves are the most important raw material of cigars. Studying the genomic information of cigar tobacco is conducive to improving cigar quality from the perspective of genetic breeding. However, no reference genome or full-length transcripts at the genome-wide scale have been reported for cigar tobacco. In particular, anion channels/transporters are of high interest for their potential application in regulating the chloride content of cigar tobacco growing on coastal lands, which usually results in relatively high Cl^−^ accumulation, which is unfavorable. Here, the PacBio platform and NGS technology were combined to generate a full-length transcriptome of cigar tobacco used for cigar wrappers.

**Results:**

High-quality RNA isolated from the roots, leaves and stems of cigar tobacco were subjected to both the PacBio platform and NGS. From PacBio, a total of 11,652,432 subreads (19-Gb) were generated, with an average read length of 1,608 bp. After corrections were performed in conjunction with the NGS reads, we ultimately identified 1,695,064 open reading frames including 21,486 full-length ORFs and 7,342 genes encoding transcription factors from 55 TF families, together with 2,230 genes encoding long non-coding RNAs. Members of gene families related to anion channels/transporters, including members of the *SLAC* and *CLC* families, were identified and characterized.

**Conclusions:**

The full-length transcriptome of cigar tobacco was obtained, annotated, and analyzed, providing a valuable genetic resource for future studies in cigar tobacco.

**Supplementary Information:**

The online version contains supplementary material available at 10.1186/s12870-021-03091-6.

## Background

Tobacco (*Nicotiana tabacum* L.),which belong to the family of Solanaceae family, is a major economically important crop species that is widely cultivated [1]. Based on the curing method used and agronomic characteristics, economically important tobacco in China can be grouped into four types: flue-, sun- and air-cured tobacco and burley tobacco [2].As a kind of sun-cured tobacco, cigar tobacco is cultivated most commonly and traditionally. Cigars are combusted tobacco products consisting of filler, binder and wrapper materials, which are all derived from cigar tobacco [3]. High-quality cigar tobacco worldwide is mainly produced in Cuba, the United States, Ecuador, Indonesia and other countries. Compared with the world’s most famous cigar origin, Cuba [4], Hainan Province of China has a similar latitude, climate conditions and rich, red silty loam. Therefore, this location could be a good place in China to produce high-quality raw materials for cigar production. However, high chloride content in the soil (as can occur on islands) is unfavorable and excessive amounts of chloride absorbed and translocated to tobacco leaves hinder cigar quality. Furthermore, salt stress is also a threat to the normal growth of cigar tobacco. In order to study the molecular genetic basis of cigar tobacco and its stress tolerance for breeding purposes, it is important to obtain the nucleotide sequences of its genes. According to the NCBI database, there are three high-quality draft genomes for the main tobacco varieties, K326 (flue-cured), TN90 (burley) and Basma Xanthi (BX, oriental) [5]. To date, there have been no reports about the genome sequence of cigar tobacco.

Next-generation high-throughput sequencing (NGS), which is commonly known as second-generation sequencing, has been widely used in plant transcriptome analyses [6–8]. Although it is a powerful and economical way to get genetic information from plants even without the aid of a reference genome, the short-read lengths generated can restrict correct sequence assembly and annotation. Recently, the third-generation sequencing (TGS) platform SMRT (single molecular real-time) sequencing performed in conjunction with the PacBio Iso-Seq protocol has been used to analyze full-length transcriptomes of multiple plant species, such as *Camellia* [9], *Medicago falcate* [10], Chinese cabbage (*Brassica rapa L. ssp. pekinensis*) [11], *Crocus sativus* [12] and *Gossypium australe* [13].

In addition to nitrate (NO_3_^−^), chloride (Cl^−^) is the other important anions in plant cells. As an essential micronutrient in many plant species, Cl^−^ plays important roles in a series of cellular and biological processes, such as stomatal movement, photosynthesis, cellular osmotic pressure maintenance and disease resistance [14–16]. There are three gene families that encode plant anion channels/transporters, the *SLAC* (slow anion channel), *ALMT* (aluminum-activated malate transporter) and *CLC* (chloride channel) families [17]. *SLAC* genes participate in stress signaling, growth and development and hormone responses in *Arabidopsis*. SLAH1, 2 and 3, which are members of the SLAC family, have been reported to control nitrate and chloride loading in the root xylem [18, 19]. *ALMT* genes participate in aluminum tolerance, stomatal opening, and fruit acidity in plants. In *Arabidopsis*, the members of the AtALMT family (14 members) can be divided into four clades, and AtALMT9 is a malate-activated vacuolar chloride channel that is required for stomatal opening [20, 21].*CLC* genes also appear to be key players in the regulation of cell pressure potential, stomatal movement, and nutrient transport. In Arabidopsis, AtCLCc, AtCLCe and AtCLCg were shown to transport Cl^−^ compared with other anions [22–24].

In the present study, both NGS and PacBio Iso-Seq were applied to cigar tobacco to obtain a global overview of the transcriptome. Therefore, transcription factors (TFs) and long non-coding RNAs (lncRNAs) were also investigated. To identify anion channels/transporters contributing to the transport of chloride in cigar tobacco, *SLAC* and *CLC* gene family members were characterized. Based on RNA-Seq, the expression levels of the identified genes were analyzed, and the validity of the transcriptome sequencing data was further verified via qRT-PCR. As the first reported full-length transcriptome of cigar tobacco, the findings in this study would provide valuable resources for future studies of functional genes in cigar tobacco especially those involved in chloride metabolism.

## Results

### Reconstruction of the full-length transcriptome of cigar tobacco

**Fig. 1:**
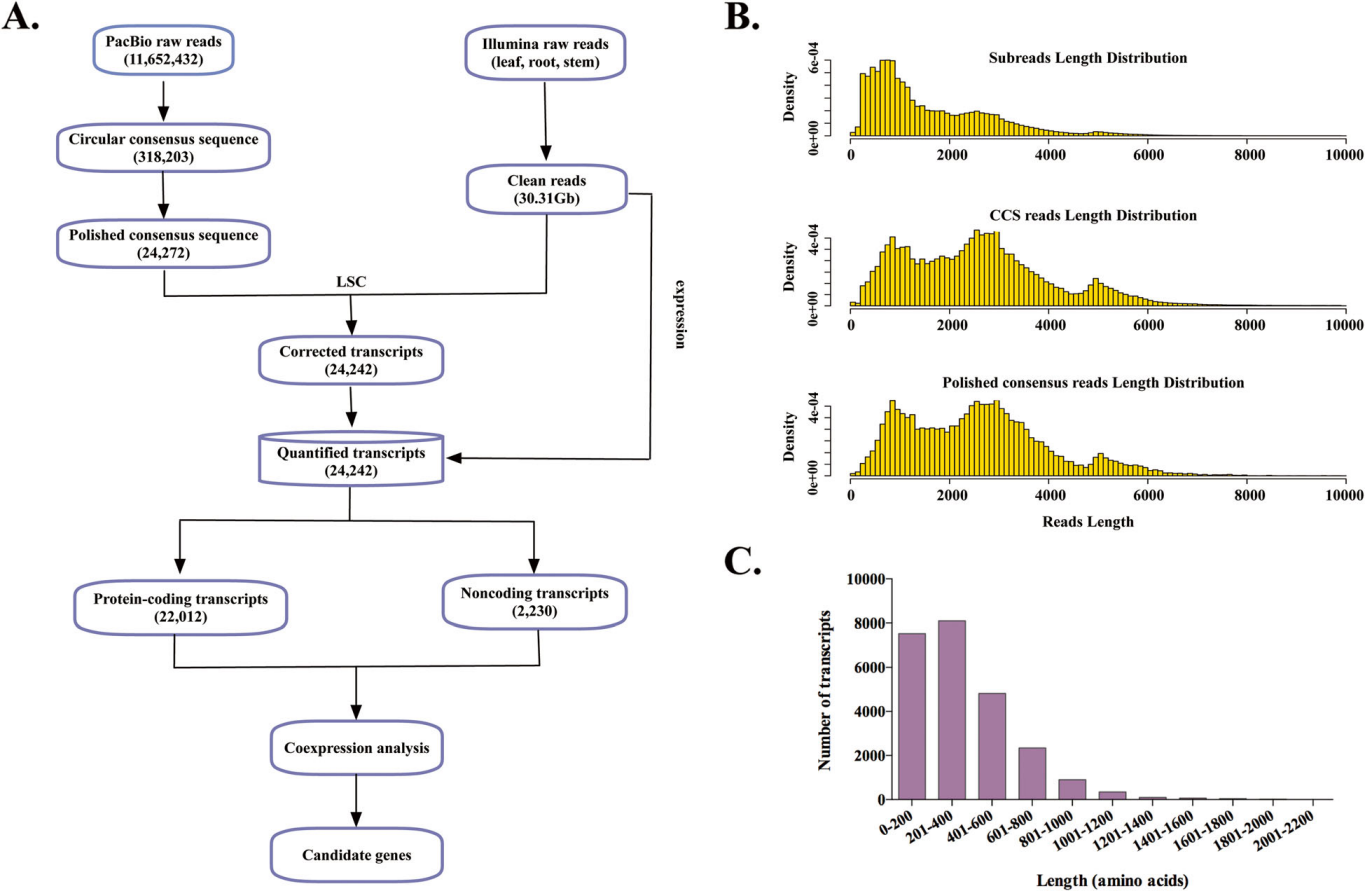
Reconstruction of the full-length transcriptome of cigar tobacco without a reference genome. **A** Pipeline used for the reconstruction of the full-length transcriptome according to PacBio Iso-seq data and NGS Illumina data. **B** Length distributions of PacBio Iso-seq data. **C** Length distribution of amino acid chains from predicted full-length ORFs

High-quality RNAs from the leaves, stems and roots of cigar tobacco were mixed in equal amounts to generate PacBio Iso-Seq libraries. The cDNAs separated according to fragment lengths of 1-2 kb, 2-3 kb, and > 3 kb. A total of 11,652,432 subreads (19-Gb) were obtained from the Pacific Bioscience RS II platform with an average read length of 1,608 bp and N50 of 2,471 bp (Table 1). According to the bioinformatics procedure shown in Fig. 1A, a total of 318,203 circular consensus sequences (CCSs) were obtained after removing primers and unwanted combinations, and the read length was improved significantly to a mean length of 2,608 bp. To polish the nonchimeric transcripts, Quiver was used and generated 24,272 polished consensus sequences, with a mean length of 2,577 bp and N50 of 3,171 bp; however, the read length distribution was not obviously different from that of the CCSs (Fig. 1B). Illumina NGS yielded 30.31 Gb of clean read data. These NGS reads were used to correct the polished consensus isoforms with LSC software, resulting in 24,242 corrected transcripts (Additional file 1: Table S1), with a mean length of 2,548 bp and N50 of 3,149 bp. Full-length ORFs were then identified, and the encoded protein sequences were predicted by ORFfinder software (https://www.ncbi.nlm.nih.gov/orffinder/). Finally, we totally predicted 1,695,064 ORFs with a mean length of 2,634 bp, while only 21,486 were identified as full-length ORFs. As shown in Fig. 1C, the length of amino acids encoded by full-length ORFs were crowded around 201–400 amino acids.

**Table 1:** Summary of reads from PacBio single-molecule long-read sequencing

	Subreads	CCS	Polished consensus reads	Corrected consensus reads
**Number**	11,652,432	318,203	24,272	24,242
**Mean length**	1,608	2,608	2,577	2,548
**N50**	2,471	3,201	3,171	3,149

### Functional annotation of transcripts

**Fig. 2:**
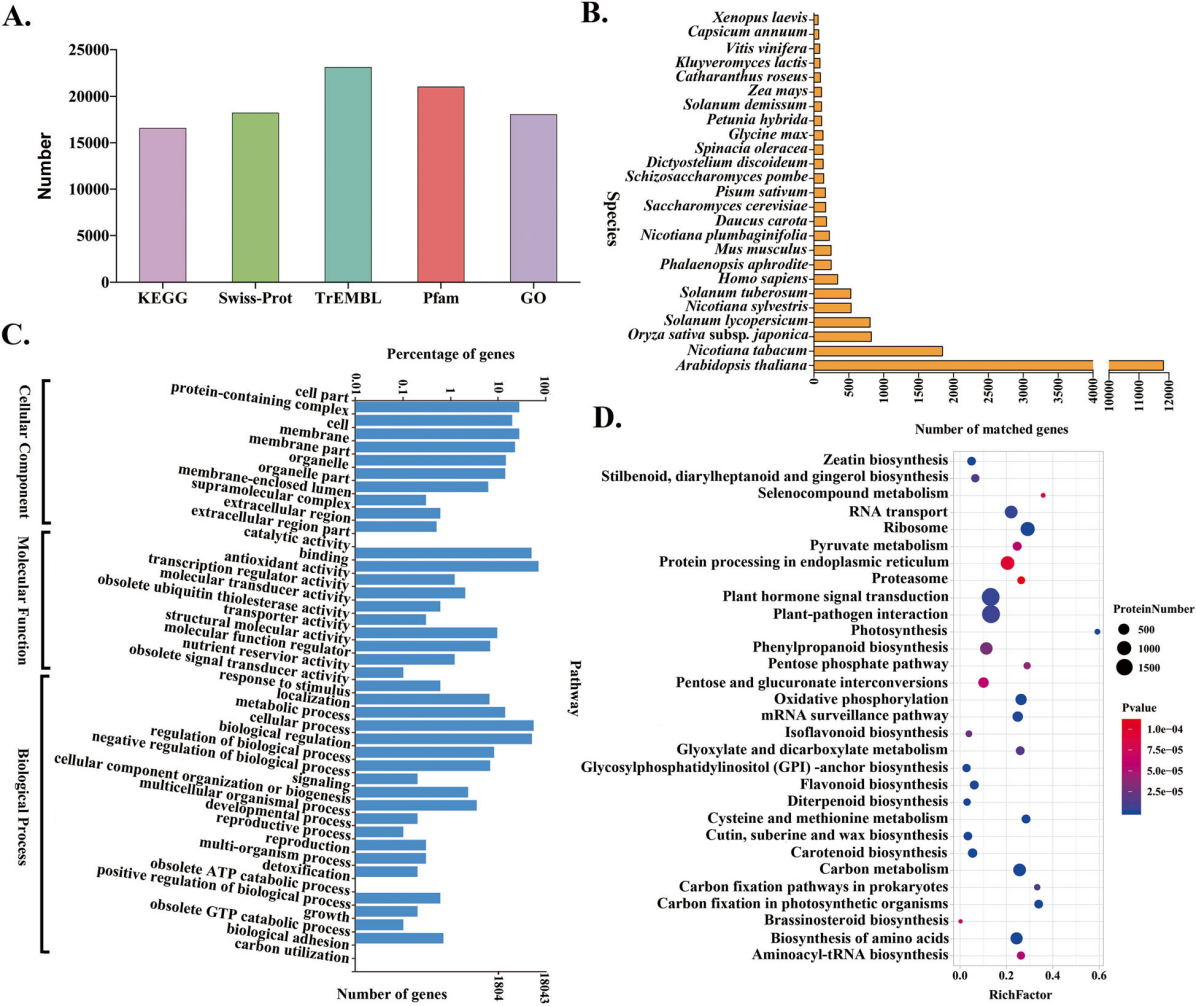
Functional annotation and classification of corrected transcripts. **A** Functional annotation of transcripts in various databases. **B** Homologous species distribution diagram of transcripts. C GO classification of all annotated transcripts. **D** KEGG pathway enrichment of transcripts. KEGG, Kyoto Encyclopedia of Genes and Genomes; GO, Gene Ontology

All 24,242 corrected transcripts were functionally annotated by searching the KEGG, Swiss-Prot, TrEMBL, Pfam and GO databases, and a total of 23,104 transcripts (95.6 % of the total transcripts)were annotated (Fig. 2A). Homologous species were analyzed by comparing their transcript sequences to those within the Swiss-Prot database, and the results showed that the top six species with the most transcripts identified were *Arabidopsis thaliana* (11,821 unigenes), *Nicotiana tabacum* (1,844 unigenes), *Oryza sativa* subsp. *japonica* (822 unigenes), *Solanum lycopersicum* (805 unigenes), *Nicotiana sylvestris* (534 unigenes) and *Solanum tuberosum* (531 unigenes) (Fig. 2B). To further functionally classify these transcripts, GO term analysis was performed (Fig. 2C; Additional file 2: Table S2). In total, 18,043 transcripts were assigned GO terms, which could be classified into three major categories (biological processes, cellular components and molecular functions). In the cellular component (CC) category, “cell part”(GO:0044464) and “cell” (GO: 0005623) were the two most prevalent functional terms (Additional file 3: Figure S1); in the molecular function (MF) category, “catalytic activity” (GO: 0003824) and “binding” (GO: 0005488) were the two most functional terms (Additional file 4: Figure S2); and in the biological process (BP) category, “metabolic process” (GO: 0008152) and “cellular process”(GO: 0009987) were the two most functional terms (Additional file 5: Figure S3). KEGG pathway enrichment provides classifications that are valuable for studying the complex biological functions of genes. As shown in Fig. 2D, several pathways, such as “plant hormone signal transduction”, “plant-pathogen interaction” and “phenylpropanoid biosynthesis” were highly enriched (Additional file 6: Table S3). Taken together, these results indicated that secondary metabolism in cigar tobacco is active.

### Identification of transcription factors

**Fig. 3:**
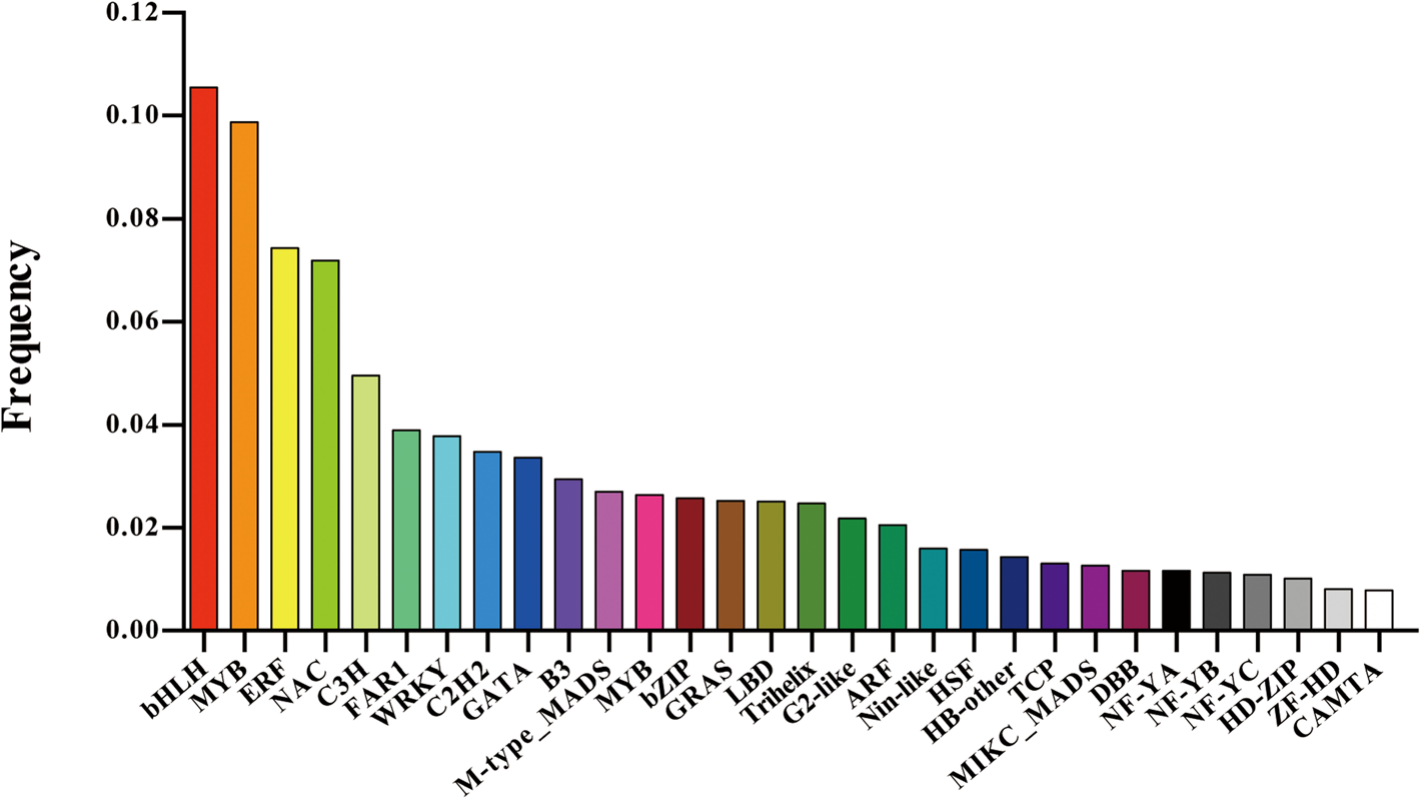
Top 30 families of identified TFs in cigar tobacco

Transcription factors (TFs) are proteins that regulate gene expression through binding to specific DNA. In general, TFs participate in lots of biological processes, such as plant growth and stress responses [25]. A total of 7,432 TFs belonging to 55 different families were annotated by BLAST using the TF database. The top 30 families identified are shown in Fig. 3. The largest TF family was the bHLH (10.55 %) family, followed by the MYB (9.87 %), ERF (7.41 %) and NAC (7.18 %) families. We further identified tissue specific TFs among three different organs (roots, stems and leaves). The majority of TFs presented a tissue-specific expression pattern on the basis of their expression profiling in our data (Additional file 7: Figure S4). Interestingly, most of the TFs were highly expressed in the roots, in which three *bHLH25* transcirpts (*transcript_16037*, *transcript_11421* and *transcript_9016*) were found to be specifically expressed. The numerous TFs identified here obviously serve as abundant resources for further research on specific TFs regulating salt stress responses or chloride transport in cigar tobacco.

### Identification of lncRNAs

**Fig. 4:**
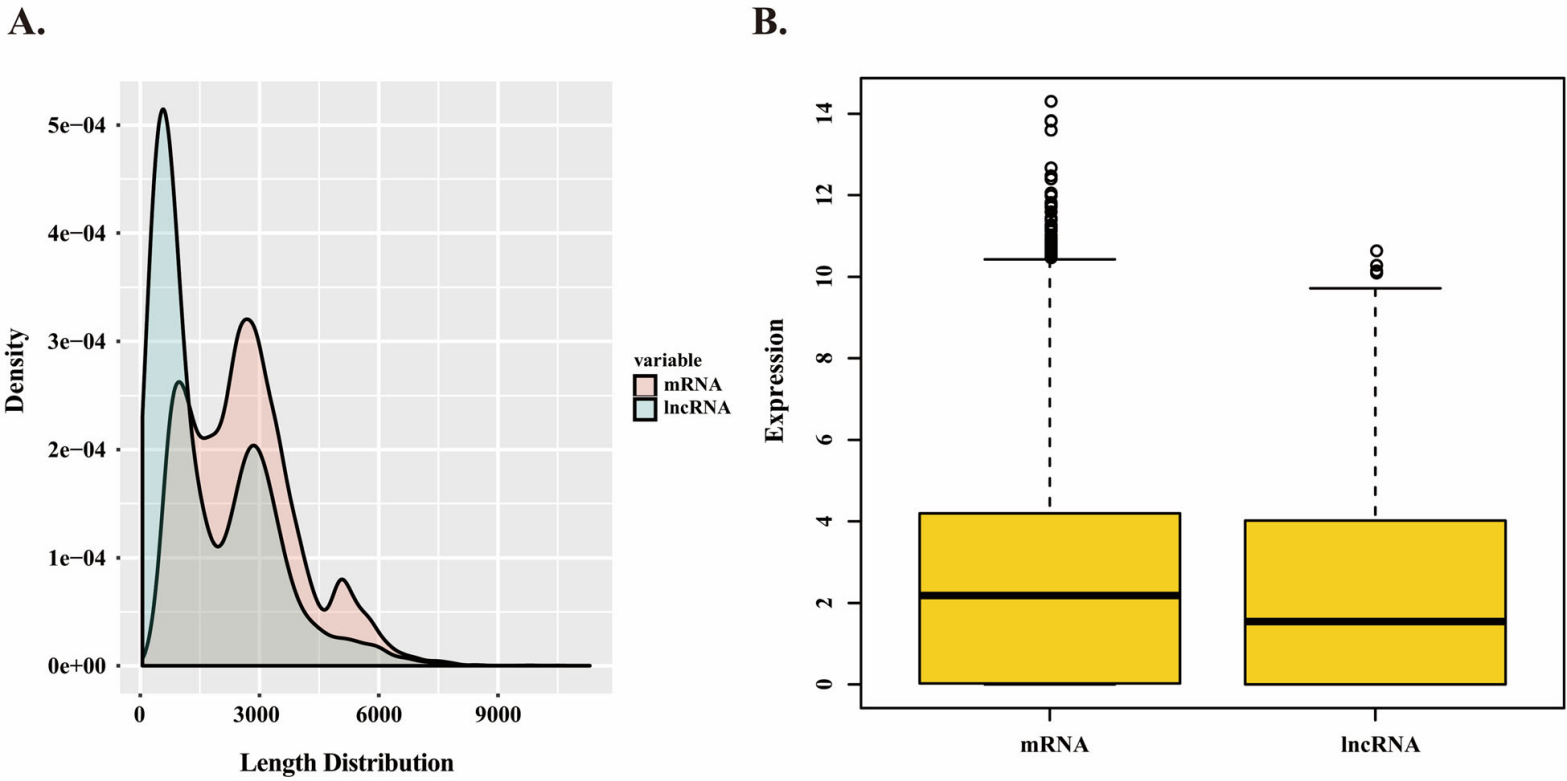
Identification of lncRNAs in cigar tobacco. **A** Density and length distributions of mRNAs and lncRNAs. **B** Expression comparison between mRNA and lncRNAs

Constituting another important component of the transcriptome, lncRNAs are shown playing various roles in regulating some biological processes [26]. Here, the CPC (Coding Potential Calculator) and Pfam (Protein family) database were used to predict lncRNAs from the PacBioIso-Seq isoforms. And totally 2,230 lncRNAs in cigar tobacco (Additional file 8: Table S4, Fig. 4A) were identified. The length of lncRNAs varied from 55 to 7,697 bp, and most (> 80 %) of them had a length ≤ 3,000 bp. Meanwhile, the mean length was 1,696 bp, which was much shorter than the coding isoforms (2,634 bp). The functions of these lncRNAs, especially the unannotated lncRNAs, need to be further characterized. Moreover, the expression of lncRNAs was lower than that of mRNAs (Fig. 4B).

### Analyses of tissue-enriched transcripts

To analyze the expression of transcripts enriched in the various tissues, a total of 9 RNA libraries were generated from the 3 different cigar tobacco tissues, with three biological replicates included. Quality-controlled RNA-seq reads from all the three organs (leaves, stems and roots) of the cigar tobacco plants were mapped to transcripts. Expressed transcripts were defined as those with an average FPKM (log2) greater than 2. We detected 12,017, 15,060, and 13,859 protein-coding transcripts in the leaves, stems, and roots, and 10,343 transcripts were expressed in all the sampled tissues (Additional file 9: Table S5), which functions like “housekeeping” genes. As expected, the 10,343 ubiquitously expressed transcripts were enriched in basic cell biological and metabolic processes according to GO enrichment analysis, including GO terms such as “organonitrogen compound metabolic and biosynthetic process”, “metabolic process”, “cellular metabolic process”, and “primary metabolic process” (Additional file 10: Table S6). Additionally, the ubiquitous category also included the terms “intracellular part”, “organelle”, “ribonucleo protein complex”, and “mitochondrial part”.

**Fig. 5:**
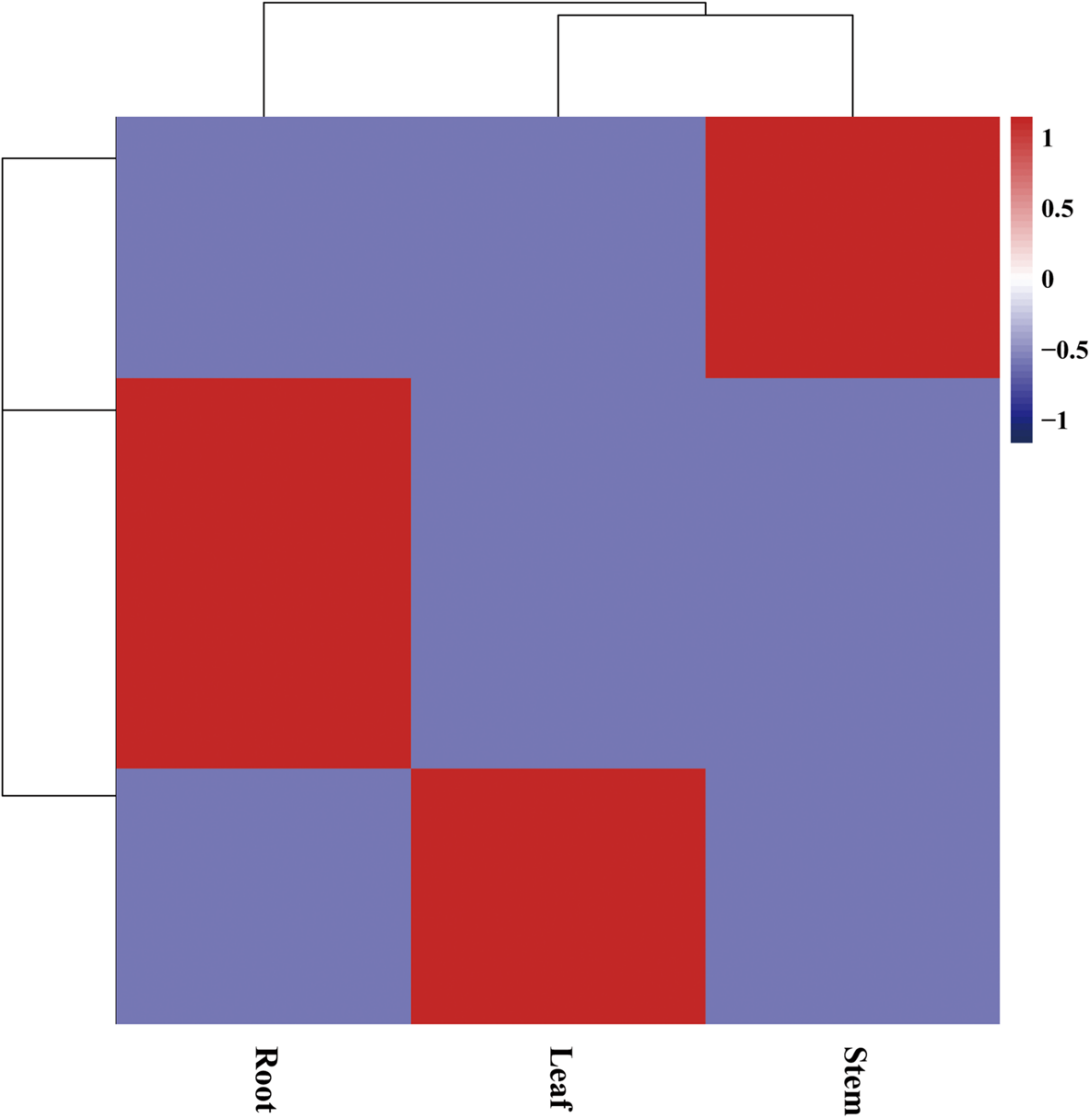
Expression heatmap of tissue-enriched transcripts in the roots, leaves, and stems

Tissue-enriched transcripts were identified in all of the three tissues. In total, 573, 374 and 383 transcripts showed tissue-specific expression patterns (Fig. 5) in the roots, leaves and stems, respectively. Pathway enrichment showed that tissue-specific transcripts were enriched in particular molecular functions that vary with tissues (Additional file 11: Table S7). For example, the transcripts enriched in root tissue were associated with nicotinate and nicotinamide metabolism (shown in the third line, sheet of “root_KEGG” in Additional file 11: Table S7), which is consistent with nicotine biosynthesis in the roots. Commonly, transcription factor may regulate gene expression using specific cis element. We did motif enrichment analysis for these tissue specific expressed genes (Additional file 12: Figure S5). Several interesting motifs for known transcription factors were found for root/leaf/stem. For example, the motif (ID: C2C2dof tnt. OBP3 col an m1) of OBP3 (also known as OBF-binding protein 3) that encode a nuclear localized Dof domain were enriched for root specifically expressed genes.

### Characterization of the transcripts of anion channels/transporters in cigar tobacco

Plant cells contain several inorganic anions, such as NO_3_^−^, Cl^−^ and SO_4_^2−^ [27]. The identification of anion channels/transporters is important to understand the regulatory mechanisms of these anions. Here, we attempted to identify members of three main anion transporter/channel families (SLAC, CLC and ALMT) using full-length transcriptomic data of cigar tobacco. In total, 22 *CLC* genes and 8 *SLAC* genes were ultimately obtained, while no *ALMT* genes were identified. Compared with those in other plant species (Table 2), the numbers of *CLC*, *SLAC* and *ALMT* genes identified in cigar tobacco are limited, especially for the *ALMT* family, which may be due to the lack of detection resulting from low expression of defects during sequence assembly in the absence of genomic information.

**Fig. 6:**
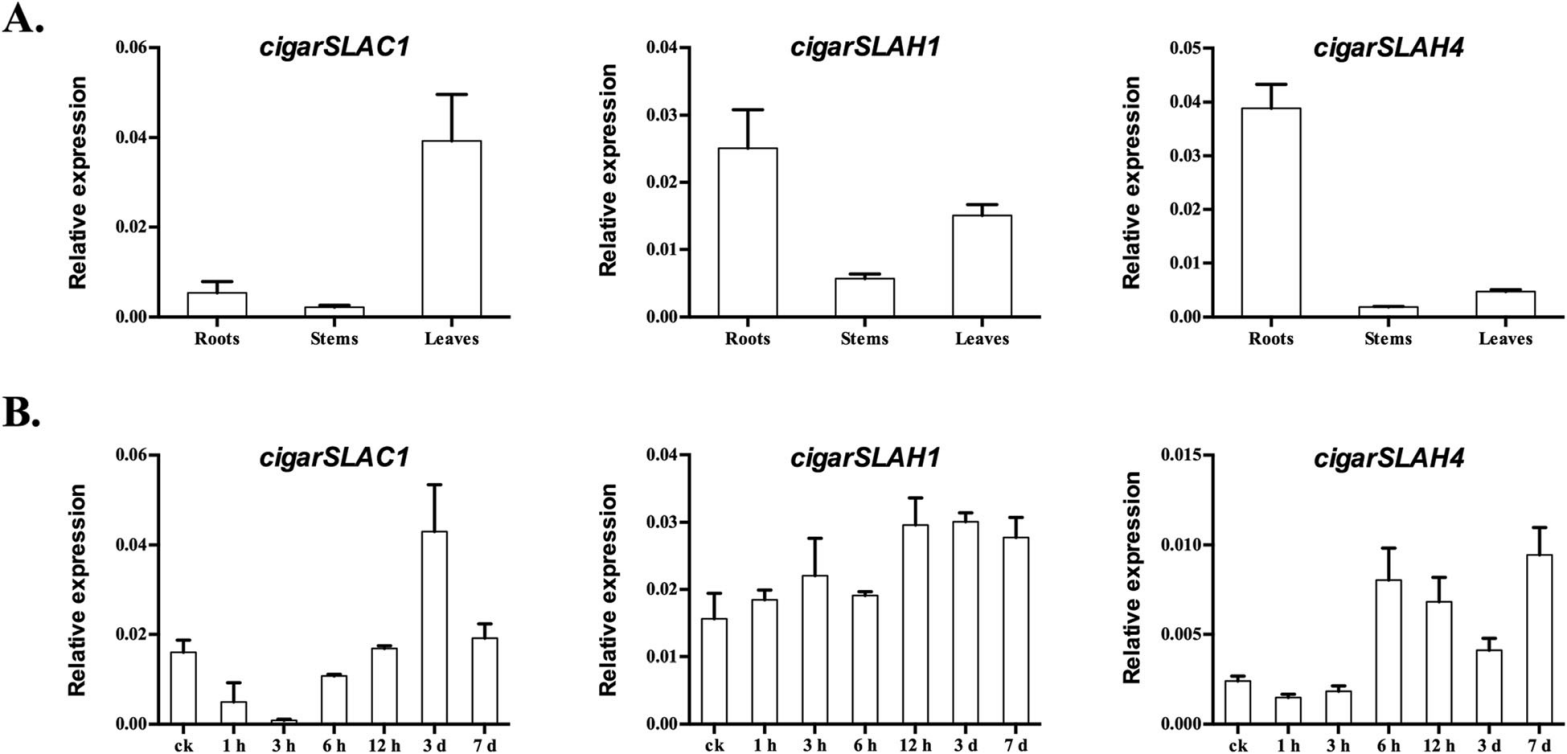
Expression patterns of *cigarSLAC1*, *cigarSLAH1* and *cigarSLAH4*, as revealed by qRT-PCR. **A** Expression patterns of the three genes in the roots, stems and leaves. **B** Expression patterns of the three genes in the roots of plants under salt stress

For the SLAC family, three members, *cigarSLAC1*, *cigarSLAH1* and *cigarSLAH4*, were successfully cloned and characterized via qRT-PCR (Fig. 6A). *cigarSLAC1* and *cigarSLAH4* showed tissue-specific expression patterns; these genes were expressed predominantly in the leaves and roots, respectively. The tissue expression of *cigarSLAH1* ranked as follows: roots > leaves > stems. To determine whether these three *cigarSLAC* genes are involved in the salt stress response, the expression levels in the roots of plants under salt stress were measured (Fig. 6B). *cigarSLAC1* showed the highest expression after salt stress for 3 days, and the expression of *cigarSLAH4* was upregulated under salt stress after 6 h, while no significant changes were observed for *cigarSLAH1*. It would be interesting to further study the function of *cigarSLAC1* and *cigarSLAH4* to reveal the mechanism underlying cigar resistance or adaptation to salt stress.

**Table 2:** Numbers of *CLC*, *SLAC* and *ALMT* genes in different plant species

Species	Gene Family		
	***CLC***	***SLAC***	***ALMT***
***Nicotiana tabacum*(cigar tobacco)**	**22**	**8**	**0**
***Nicotiana sylvestris***	**20**	**15**	**22**
***Nicotiana tomentosiformis***	**16**	**13**	**22**
***Nicotiana tabacum*(BX)**	**36**	**18**	**33**
***Nicotiana tabacum*(K326)**	**10**	**13**	**27**
***Nicotiana tabacum*(TN90)**	**35**	**18**	**44**
***Arabidopsis thaliana***	**6**	**5**	**10**
***Oryza sativa*(rice)**	**10**	**9**	**10**
***Solanum lycopersicum*(tomato)**	**5**	**7**	**16**

**Fig. 7:**
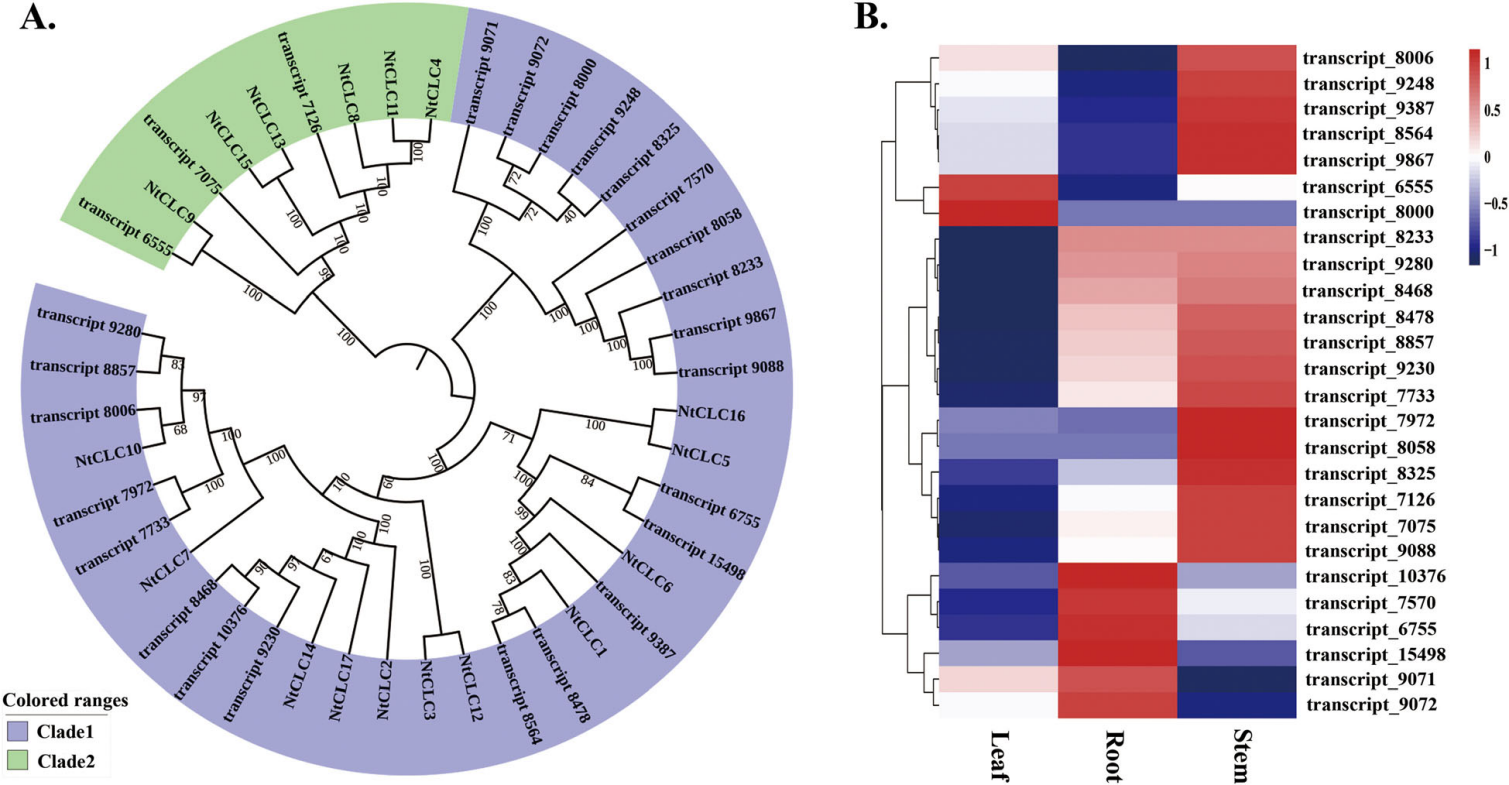
Phylogenetic analysis of *cigarCLCs* (**A**) and the expression heatmap of *cigarCLCs* in different tissues (**B**)

For the CLC family, a phylogenetic tree was constructed together with the protein sequences of known CLC members in flue-cured tobacco (K326) using the neighbor-joining algorithm (Fig. 7A). All the CLCs from cigar tobacco clustered into two clades, which is similar to the clustering results of K326 [28]. According to RNA-seq, most of the *cigarCLC* genes showed a higher expression in stems, and only two and six members showed clearly higher expression in the leaves and roots, respectively (Fig. 7B).

## Discussion

As an important part of cigars, the wrapper affects the quality of cigars directly. To date, there have been no reports about the genome sequence of cigar tobacco. Recently, along with the development of third-generation sequencing technology, full-length transcriptome sequencing has been used in species without a reference genome sequence because of its advantages of scale, sampling depth, transcript completeness and cost.

In this study, the first comprehensive full-length transcriptome sequences are presented for cigar tobacco using PacBio Iso-seq technology, the most popular platform for third-generation sequencing. After correction by the use of Illumina sequencing reads, we generated 24,242 transcripts, with a mean length of 2,577 bp and N50 of 3,171 bp. Based on these highly accurate transcripts, up to95.6 % of the transcripts were annotated in five functional databases (KEGG, Swiss-Prot, TrEMBL, Pfam and GO), which suggested that this study generated a very large number of cigar tobacco genes. GO annotation analysis showed that “cell part”, “cell”, “catalytic activity”, “binding”, “metabolic process” and “cellular process” composes the majority of subcategories. KEGG annotation analysis showed that “plant hormone signal transduction”, “plant-pathogen interaction” and “phenylpropanoid biosynthesis” are the top three pathways with the greatest abundance unigenes. Both GO and KEGG analyses indicated that the organs of cigar tobacco plants were undergoing active cell metabolism to accumulate necessary nutrients for continued growth and development.

Long-read transcriptome sequencing can provide genetic information for transcriptional and posttranscriptional regulation analyses (those involving TFs, lncRNAs, and so on). TFs can bind to cis-regulatory elements in promoters or enhancers and thus regulate transcriptional initiation. In total, 7,432 unigenes encoding TFs belonging to 55 different families were characterized based on the current cigar tobacco transcriptome. Among them, bHLH, MYB, ERF and NAC were the most represented TF families. In addition, most of the TFs showed tissue-specific expression, and of them, root-specific expression was in majority. The bHLH family is one of the largest classes of plant TFs, and their members participate in the regulation of a series of essential biological processes such as transcriptional activation, flavonoid biosynthesis and stress responses[29]. In fact, the participation of TFs in plant response to salt stress were well reported, such as MYC2, a bHLH transcription factor, imparting salt intolerance by regulating proline biosynthesis in Arabidopsis [30], GmMYB118 increasing tolerance to drought and salt stress in transgenic Arabidopsis [31], PvERF35 promoting salt stress tolerance in tobacco plants [32], and NAC positive function in crop salt tolerance [33–35].Obviously, the TFs, especially those root-specific TFs, identified from our transciptome data would promote the discovery of transcription factors in response to salt stress in cigar tobacco.

lncRNAs are universal in plants and are involved in various biological regulatory processes [26]. lncRNAs are often regarded as RNA transcripts with lengths greater than 200 bp but not coding for a protein. Recently, several lncRNAs have been characterized in the plant salt stress response [36, 37]. Although, some functional predictions of salt-responsive lncRNAs in different plant species have been reported, whether lncRNAs can regulate chloride uptake or transport in tobacco needs further study. Here, we used three tools to identify lncRNAs from our PacBio Iso-Seq data and identified a total of 2,230 lncRNAs in cigar tobacco. The mean length of the lncRNAs (2,115 bp) was shorter than that of protein-coding mRNAs (2,634 bp), which is consistent with the results of previous research [38].

The study of tissue-specific genes can provide insights into tissue development and evolution. Here, we performed RNA-seq profiling of genes expressed in three tissues of cigar tobacco. In total, 12,981 (leaf), 14,240 (stem) and 13,906 (root) protein-coding transcripts were detected. In addition to the 11,331 “housekeeping” transcripts that were expressed in all sampled tissues, 554, 471 and 894 transcripts were identified as being enriched in the leaves, stems and roots, respectively. Moreover, GO analysis showed that leaf tissue-enriched genes were associated with metabolic processes and both the stem tissue-enriched and root tissue-enriched genes were associated with binding.

Anion channels/transporters are important to a series of physiological functions in plants (for example, cell signaling, plant nutrition, metal tolerance and so on) [17]. In the past few years, the members of three gene families, *SLACs*, *ALMTs* and *CLCs*, have enriched and improved the awareness of the various types of genes encoding plant anion channels/transporters. Slow anion (S-type) channels play key roles in plant anion (such as chloride and nitrate) transport [18]. In Arabidopsis, there are five members in the SLAC family: AtSLAC1, AtSLAH1, AtSLAH2, AtSLAH3 and AtSLAH4 proteins. Among them, SLAH1, 2 and 3 have been reported to control nitrate and chloride loading of the root xylem [39]. In particular, the *AtSLAH1* gene showed a great potential to apply in engineering salinity-tolerant plants [19]. Previously, we cloned the *SLAH1* gene in K326 and found that the expression of *SLAH1* was down-regulated under salt stress, which indicated its important role in the response to salt and the absorption of chloride in tobacco (data not shown). In this study, we cloned three SLAC family members: *cigarSLAC1*, *cigarSLAH1* and *cigarSLAH4*. The expression of these three genes in different tissues (roots, stems and leaves) and response to salt stress were also checked by qPCR. It was reported that *AtSLAC1* is the key player in the regulation of stomatal closure [40]. Here, *cigarSLAC1* showed a higher expression in leaves, but whether it could regulate the stomatal closure in cigar tobacco needs more experimental confirmation. As *cigarSLAH1* showed no significant tissue-expression pattern and the expression was almost no difference after salt stress, it was a question whether *cigarSLAH1* could modulate shoot Cl^−^ accumulation in cigar tobacco as *AtSLAH1*. It was interesting that *cigarSLAH4* exhibited a root-specific expression pattern, and was significantly induced after 6 h of salt treatment. Since the function of *AtSLAH4* is still not clear, our data provide a preliminary role of *cigarSLAH4* under salt stress. For the CLC family, it has been reported that few of the *CLC* genes take part in plant salt tolerance and function by mediating Cl^−^ transport across the tonoplast [41–43]. In a previous study, we identified and analyzed members of the CLC family in flue-cured tobacco (K326) [28], and 17 *NtCLC* members were characterized and divided into two clades according to a phylogentic analysis. Moreover, *NtCLC2* and *NtCLC13* were indicated to play roles in chloride transport or metabolism in tobacco. In this study, 26 *cigarCLC* transcripts were obtained based on PacBio Iso-Seq data. Moreover, according to the phylogenetic tree, the *cigarCLCs* could be divided into two clades, similar to the division of *NtCLCs*. We also noticed that *transcript_7126* was closely related to *NtCLC13*, and that *transcript_8468*, *transcript_9320* and *transcript_10376* were closely related to *NtCLC2*. Whether these four transcripts function in chloride metabolism needs further study.

## Conclusions

In this study, we reconstructed and analyzed the full-length transcriptome of cigar tobacco by combining PacBio SMRT sequencing technology and NGS Illumina sequencing. In total, 24,257 transcripts, 7,432 transcription factors and 2,230 lncRNAs were identified and analyzed. Furthermore, the gene families related to anion channels/transporters, including the SLAC and CLC families were identified and characterized in cigar tobacco. These results could provide important information and candidate genes for future cigar biological studies, especially given that there is no reference genome of cigar tobacco.

## Methods

### Plant material and growth conditions

Cigar tobacco (*Nicotiana tabacum* L., ‘BES NO H382’) seeds, which were donated by the Hainan Cigar Research Institute of the Hainan Company of CNTC, were sown in nutrition-enriched media until germination. Afterward, the plants were grown in plastic pots under a 16 h light photoperiod at 28 ^o^C during the day and at 23 ^o^C at night. Three individual lines were selected and used for further experiments. When the plants grew to the six-leaf stage, fresh leaves, stems and roots were harvested, frozen in liquid nitrogen, and then stored at -80 ^o^C.

For salinity stress, plants at the six-leaf stage were first transferred to a 1/2-strength MS solution. One week later, the plants were then treated with 300mM NaCl in 1/2-strength MS solution for 7 days. The roots were removed from the plants at 1 h, 3 h, 6 h, 12 h, 3d and 7d after salt stress. The roots were then rinsed free of 1/2-strength MS solution, patted dry, and waiting for RNA extraction.

### Library preparation, SMRT sequencing and Illumina RNA-Seq sequencing

Total RNA from each sample was isolated using a SuperPure Plantpoly RNA Kit (Gene Answer). RNase-free DNase I (Gene Answer) was used to remove any contaminating DNA. The purity and concentration of total RNA was determined using a NanoDrop 2000 instrument (Thermo) [28]. Five micrograms of total RNA (all three RNAs pools were mixed equally) was used as an input for Clontech SMARTer reactions.

BluePippin™(Sage Science, Beverly, MA) was used to perform the size fractionation and selection and finally three size ranges (1-2 kb, 2-3 kb, and > 3 kb) were obtained. For cDNA library construction, a Pacific Biosciences DNA Template Prep Kit 2.0 was used to generate the three SMRT bell libraries. Then, SMRT sequencing was performed on the Pacific Bioscience RS II platform. Figure 1A lists the workflow for the whole PacBio Iso-seq data processing procedure.

RNA samples from the roots, stems and leaves were used for Illumina library construction and sequencing. The methods of library construction, sequencing and data processing were the same as those described previously [28]. The quality and size of the cDNA libraries for sequencing were checked using an Agilent 2200 TapeStation system (Agilent). The libraries were run individually on single lanes for 100 cycles (paired-end) on Hiseq™ 2000 (Illumine Inc.).

### Quality filtering and error correction

Briefly, ccs (https://github.com/PacificBiosciences/ccs) was firstly used to generate circular consensus sequence (CCS) for each sequencing run. And primers and unwanted combinations for CCSs were removed. Lima (https://github.com/pacificbiosciences/barcoding) was then applied to orient each sequence to the 5’-3’ direction. The final polished clustered sequences for each transcript were created in polish mode, which were corrected by LSC [44, 45] correction tool with default parameters using NGS reads. BUSCO [46] was used to explore the completeness according to conserved ortholog content.

For the NGS reads, Trimmomatic (v0.30) (https://www.bioinformatics.babraham.ac.uk/projects/trim_galore/) was used to remove the adapter sequences and the low-quality reads ofRNA-Seq reads. Then, left reads were mapped to reference transcripts from PacBio using Bowtie2. Expression levels for transcripts were assessed via FPKM (fragments per kilobase per million reads) values by Salmon [47].

### Functional annotations

Functional annotations of all transcripts were performed using BLAST15 (version 2.2.26) [48] to compare transcripts against those in the SwissProt (a manually annotated, nonredundant protein database) [49], GO (Gene Ontology) [50], Pfam (a database of conserved protein families or domains) [51], TrEBML (a supplementary database of SwissProt) [52] and KEGG (Kyoto Encyclopedia of Genes and Genomes) [53] databases, with an E-value threshold of 10^− 3^. The functional information was assigned to the best-matched sequence. GO annotations and KEGG pathways were subsequently searched via KOBAS v2.0 [54].

### Prediction of lncRNAsand ORFs

LncRNAs (long noncoding RNAs) are usually designated as RNA transcripts which cannot encode proteins but with a length more than 200 bp. The CPC [55] and Pfam were used to distinguish the coding potential.

To identify ORFs, the potential coding sequences were predicted by using the ORFfinder software (http://transdecoder.sf.net). The transcripts containing not only complete ORFs but also 5’- and 3’-UTRs (untranslated regions) were regarded to full-length transcripts. And the putative protein sequences were predicted by CPC.

### Analysis of transcipts enriched in tissues

Transcripts enriched in tissues (the expression level in one particular tissue is significantly higher compared to all other tissues) were identified in each type of tissue based on the Roku criteria with a cutoff of 1. Motif enrichment of the tissue specific expressed genes was analyzed by AME (https://meme-suite.org/meme/tools/ame).

### qRT-PCR

Total RNA samples were isolated from cigar tobacco roots and leaves for qRT-PCR with a SuperPure Plantpoly RNA Kit (Gene Answer) as previously described. First-strand cDNA was then reverse transcribed using a Transcriptor First Strand cDNA Synthesis Kit (Roche). A LightCycler® 96 Real- Time PCR System (Roche) was used to perform the qPCR amplification reactions. All the samples were analyzed, and gene expression levels were normalized to 26 S. The primers used in this study were designed according to the PacBio-seq isoform sequences, which are listed in Additional file 13: Table S8. The qRT-PCR amplification conditions were as follows: incubation at 95 °C for 10 s, followed by 40 cycles of 95 °C for 10 s, 60 °C for 20 s, and 72 °C for 20 s. The specificity of the primer amplifications was tested using melting curve analysis, and the PCR products were verified by sequencing.

### Data availability

Raw sequence data reported in this study have been deposited in the Genome Sequence Archive [56] of the National Genomics Data Center [57], Beijing Institute of Genomics (BIG, China National Center for Bioinformation), Chinese Academy of Sciences, under accession numberCRA003020, and are publicly accessible at https://bigd.big.ac.cn/gsa.

## Supplementary Information

**Additional file 1: Table S1. **TPM for corrected transcripts based on NGS datasets.

**Additional file 2: Table S2.** GO term annotations for each transcript.

**Additional file 3: Figure S1.** Cellular components associated with annotated transcripts.

**Additional file 4: Figure S2.** Molecular functions associated with annotated transcripts.

**Additional file 5: Figure S3.** Biological processes associated with annotated transcripts.

**Additional file 6: Table S3.** KEGG pathway enrichment of annotated transcripts.

**Additional file 7: Figure S4. **Heatmap for tissue-specific transcription factors.

**Additional file 8: Table S4. **Identified lncRNAs in cigar tobacco.

**Additional file 9: Table S5. **Housekeeping transcripts in cigar tobacco.

**Additional file 10: Table S6.** GO enrichment of housekeeping transcripts.

**Additional file 11: Table S7. **GO enrichment and KEGG enrichment of tissue-specific transcripts.

**Additional file 12: Figure S5. **Motif enrichment analysis of the tissue specific expressed genes in different tissues (A. leaf; B. root; C: stem) by AME.

**Additional file 13: Table S8. **Sequences of primers used for *SLAC* transcripts.

## Data Availability

The dataset used in this study were available in the BIG Data Center at http://bigd.big.ac.cn with the accession number CRA003020.

## Abbreviations

PacBio: Pacific Biosciences
NGS: Next-generation sequencing
TGS: Third-generation sequencing
SMRT: Single Molecular Real-Time
CCS: Circular consensus sequence
ORF: Open reading frame
CDS: Coding Sequences
FPKM: Fragments Per Kilobase of transcript per Million mapped reads
GO: Gene Ontology
KEGG: Kyoto Encyclopedia of Genes and Genomes
LncRNA: Long noncoding RNA
TF: Transcription factor
SLAC: Slow anion channel
CLC: Chloride channel
ALMT: Aluminum-activated malate transporter
