# Supplementary figures and images for: Reconstruction of the full-length transcriptome of cigar tobacco without a reference genome and characterization of anion channel/transporter transcripts
Source: BMC Plant Biol. 2021 Jun 29;21:299. doi: 10.1186/s12870-021-03091-6 (PMC8240255; doi:10.1186/s12870-021-03091-6)

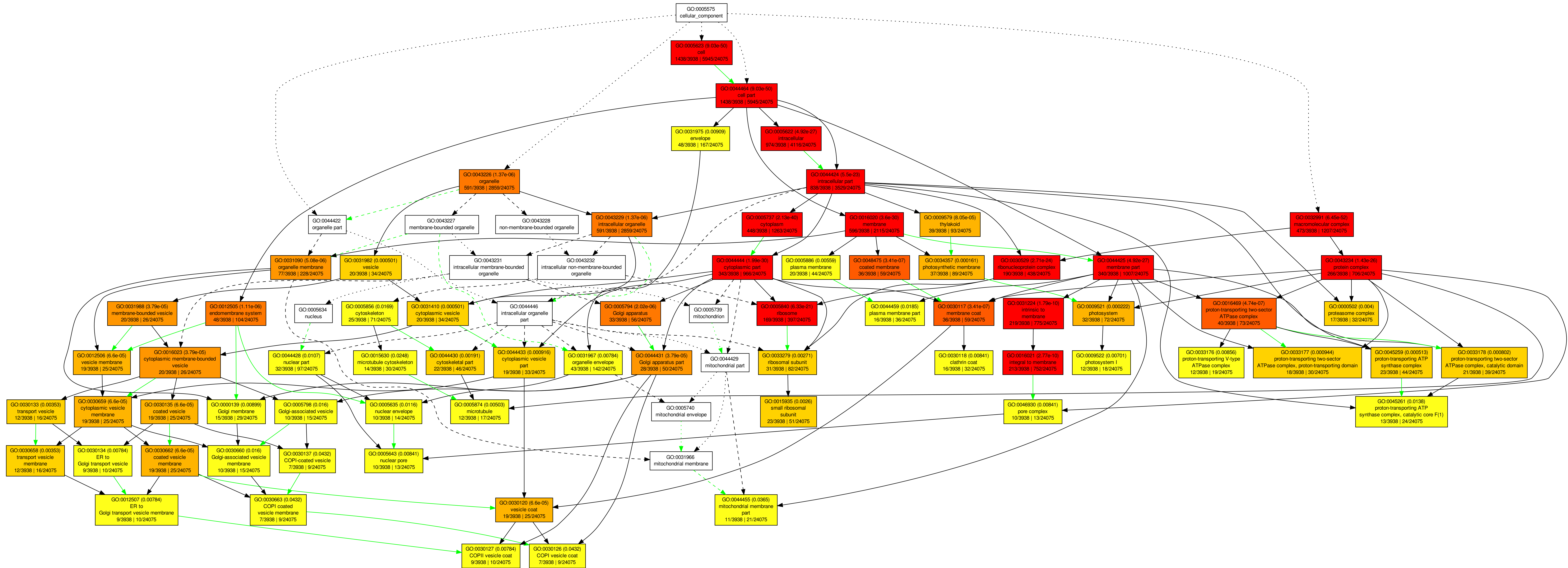

Supplement: Supplementary file 3 — Additional file 3: Figure S1. Cellular components associated with annotated transcripts. [file 12870_2021_3091_MOESM3_ESM.pdf]

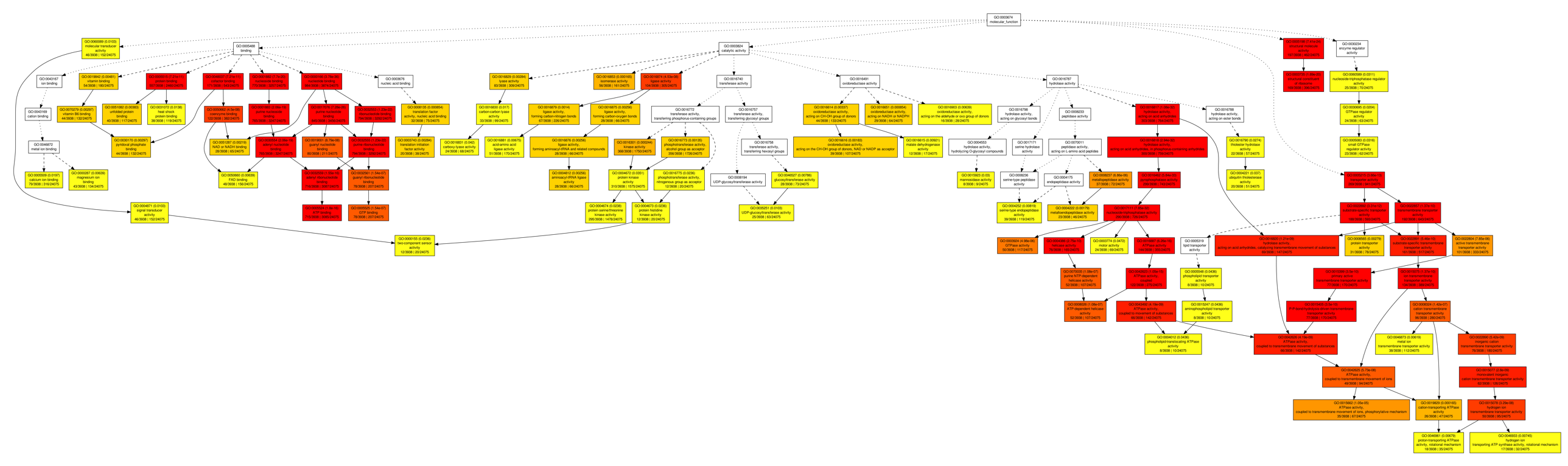

Supplement: Supplementary file 4 — Additional file 4: Figure S2. Molecular functions associated with annotated transcripts. [file 12870_2021_3091_MOESM4_ESM.pdf]

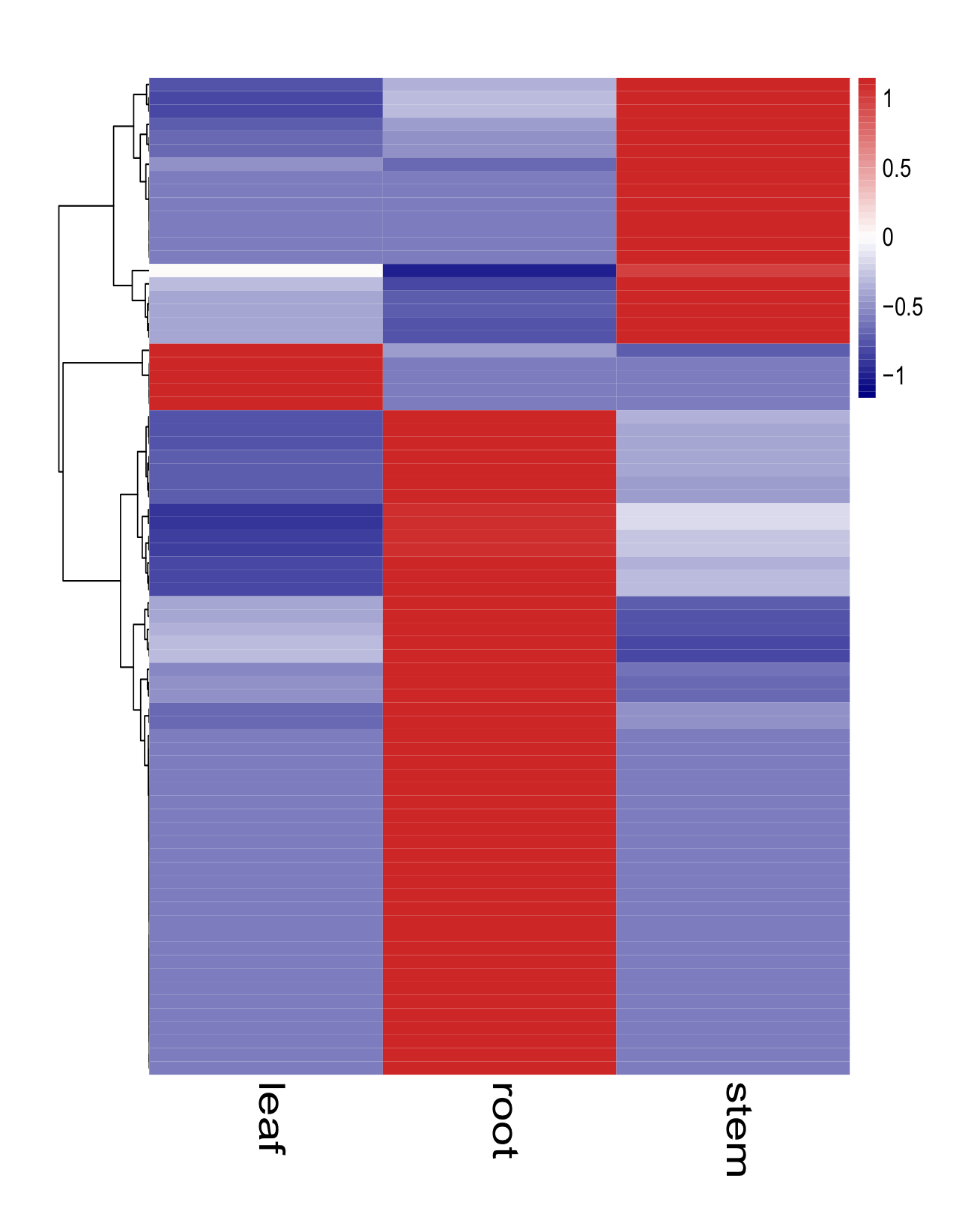

Supplement: Supplementary file 7 — Additional file 7: Figure S4. Heatmap for tissue-specific transcription factors. [file 12870_2021_3091_MOESM7_ESM.tiff]
