# Supplementary material for: Reconstruction of the full-length transcriptome of cigar tobacco without a reference genome and characterization of anion channel/transporter transcripts
Source: BMC Plant Biol. 2021 Jun 29;21:299. doi: 10.1186/s12870-021-03091-6 (PMC8240255; doi:10.1186/s12870-021-03091-6)
Supplement: Supplementary file 12 — Additional file 12: Figure S5. Motif enrichment analysis of the tissue specific expressed genes in different tissues (A. leaf; B. root; C: stem) by AME. [file 12870_2021_3091_MOESM12_ESM.docx]

A.


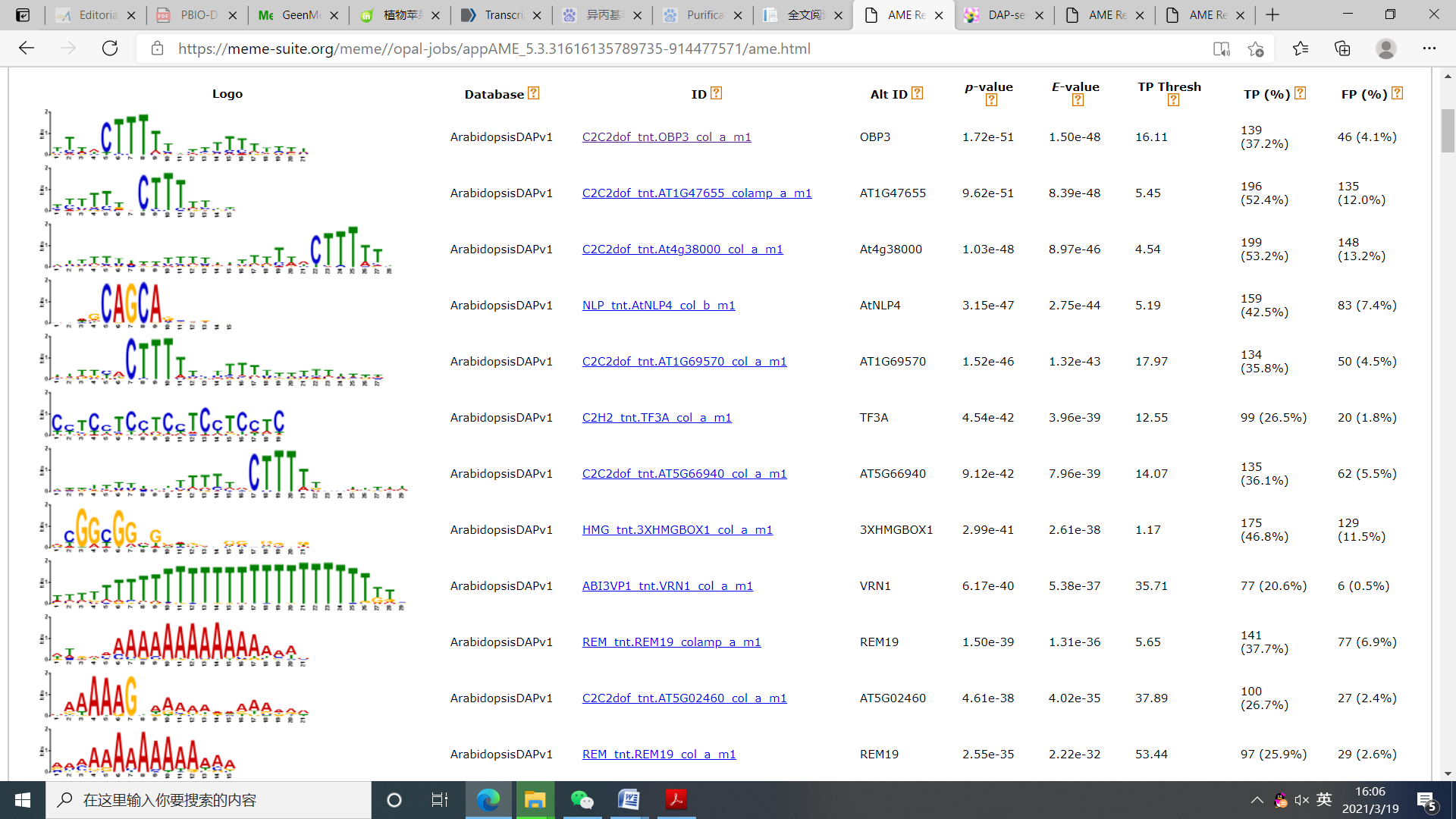


B.


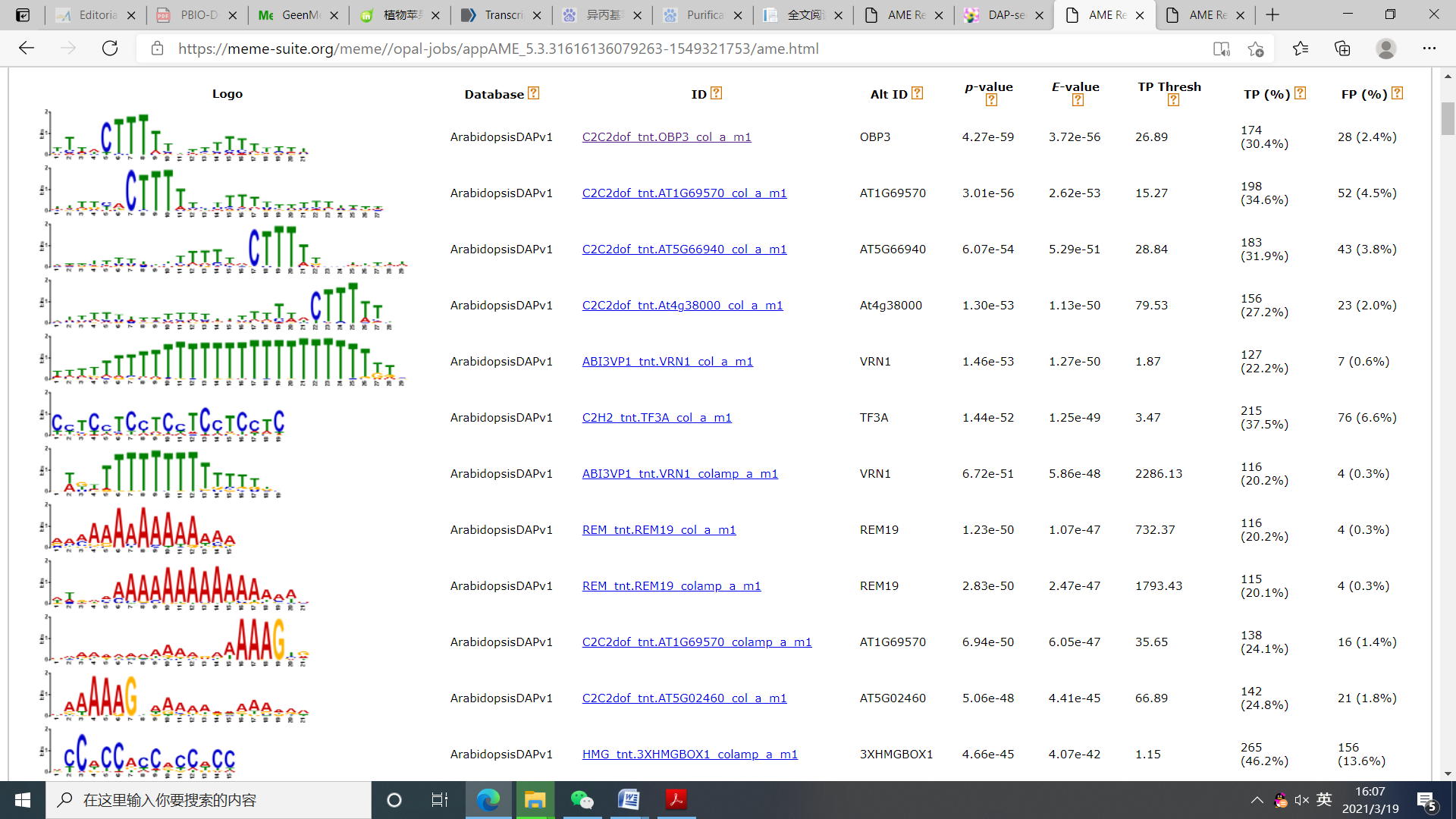


C.


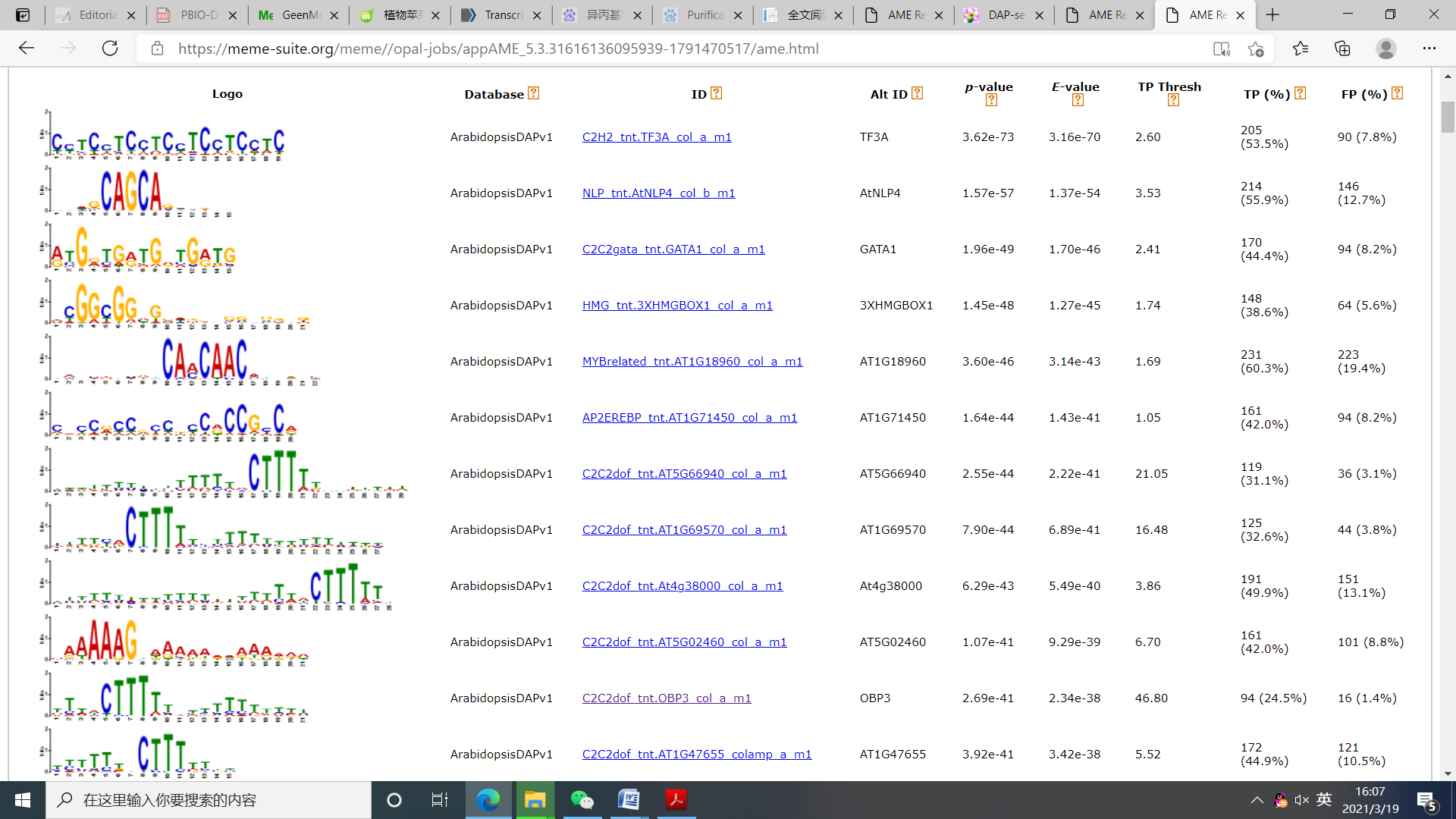


Fig S5. Motif enrichment analysis of cigar tobacco in different tissues.

Top ten motifs in three tissues, leaf (A), root (B) and stem (C).
