# Supplementary material for: Reconstruction of the full-length transcriptome of cigar tobacco without a reference genome and characterization of anion channel/transporter transcripts
Source: BMC Plant Biol. 2021 Jun 29;21:299. doi: 10.1186/s12870-021-03091-6 (PMC8240255; doi:10.1186/s12870-021-03091-6)

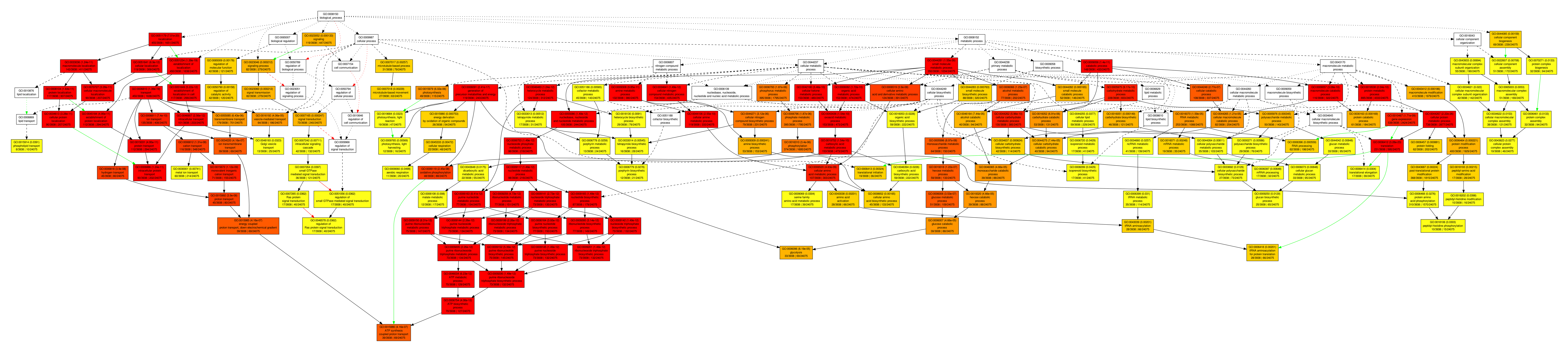

Supplement: Supplementary file 5 — Additional file 5: Figure S3. Biological processes associated with annotated transcripts. [file 12870_2021_3091_MOESM5_ESM.pdf]
